# Implementation of Safe-by-Design for Nanomaterial Development and Safe Innovation: Why We Need a Comprehensive Approach

**DOI:** 10.3390/nano8040239

**Published:** 2018-04-14

**Authors:** Annette Kraegeloh, Blanca Suarez-Merino, Teun Sluijters, Christian Micheletti

**Affiliations:** 1 INM—Leibniz Institute for New Materials, 66123 Saarbruecken, Germany; annette.kraegeloh@leibniz-inm.de; 2 TEMAS AG, 8005 Zürich, Switzerland; Blanca.SuarezM@temas.ch; 3 Public Impact, Postbus 97814, 2509 GE Den Haag, The Netherlands; t.sluijters@publicimpact.nl

**Keywords:** manufactured nanomaterials, nanomaterials functionality, Safe-by-Design, innovation, trusted environment

## Abstract

Manufactured nanomaterials (MNMs) are regarded as key components of innovations in various fields with high potential impact (e.g., energy generation and storage, electronics, photonics, diagnostics, theranostics, or drug delivery agents). Widespread use of MNMs raises concerns about their safety for humans and the environment, possibly limiting the impact of the nanotechnology-based innovation. The development of safe MNMs and nanoproducts has to result in a safe as well as functional material or product. Its safe use, and disposal at the end of its life cycle must be taken into account too. However, not all MNMs are similarly useful for all applications, some might bear a higher hazard potential than others, and use scenarios could lead to different exposure probabilities. To improve both safety and efficacy of nanotechnology, we think that a new proactive approach is necessary, based on pre-regulatory safety assessment and dialogue between stakeholders. On the basis of the work carried out in different European Union (EU) initiatives, developing and integrating MNMs Safe-by-Design and Trusted Environments (NANoREG, ProSafe, and NanoReg2), we present our point of view here. This concept, when fully developed, will allow for cost effective industrial innovation, and an exchange of key information between regulators and innovators. Regulators are thus informed about incoming innovations in good time, supporting a proactive regulatory action. The final goal is to contribute to the nanotechnology governance, having faster, cheaper, effective, and safer nano-products on the market.

## 1. Introduction

The aim of this article is to provide the authors opinion on the current, synergistic, and comprehensive approach for implementation of Safe-by-Design (SbD) into the development of Manufactured Nanomaterials (MNMs) and nano-enabled products. This comprises a brief analysis of the basic requirements for SbD, including current examples for SbD strategies, the implementation of SbD into industrial innovation processes as well as communication structures supporting the required knowledge exchange between relevant stakeholders (e.g., innovators and regulators) in that field. Combined, these three concepts will enable high tech innovations and at the same time will safeguard public interests of these innovations.

The article is partially built on opinions shared in a workshop on SbD held during the conference Nanosafety 2017 in Saarbrücken, including parts of the concepts that have been developed in the FP7 projects NANoREG and ProSafe and are still under development in the Horizon 2020 project NanoReg2.

### Safe-by-Design: A Concept for Safe Innovations

“Safe-by-Design” (SbD) is a concept that is well established in fields like building, nuclear technology, water treatment, health facilities, and occupational health and safety. It describes safety measures for the prevention of accidents, illnesses, or environmental damage that are applied during the design stage of a facility, process, practice, material, or product. Generally, SbD is not new and has been used for years in industry. Various fields have adopted and developed different, but related concepts that are first of all based on the idea to design products or processes that bear an intrinsically low risk potential, instead of confining this potential by application of protective systems. Overall, SbD has to integrate the anticipated safety impacts of materials or products into the design and production phases.

In the context of nanotechnology, SbD it is a rather novel concept, aiming at the development of functional as well as safe nanomaterials and nano-enabled products. The novelty of this concept is not due to the fact that current nanomaterials or nanoproducts are regarded as intrinsically unsafe. Rather the application of this concept requires:

- comprehensive knowledge questioning what property makes a nanomaterial or nanoproduct more or less safe;
- means to implement this knowledge in a structured way into industrial innovation processes; and
- information exchange between the involved stakeholders.

A current literature survey using Scopus (www.scopus.com) and the terms “Safe-by-Design” and “Nano” within titles, abstracts, and keywords identified 36 document results (2011–2018). 24 of these were published in 2016–2018, indicating that SbD is increasingly considered. Few of these studies demonstrate the effectiveness of their strategy to modify safety-relevant material properties (Table 1). Further studies focus on tools necessary for evaluation of the effectiveness of SbD, ranging from toxicological testing over material characterisation to hazard and risk assessment.

The implementation of SbD for MNMs is still in its infancy. It will be advanced by the proper understanding of the safety-material relationships [21]. On the other hand, understanding of the functionality-material relationships is likewise necessary in order to develop functional materials [22]. Depending on the materials properties to be exploited for a distinct application, it might not be possible to maximise both functionality and safety at the same time, as highlighted by Reinosa et al. [7].

## 2. Design of Safe Nanomaterials and Nanoproducts

Nanomaterials are distinguished by specific properties, e.g., magnetic, optical, or chemical properties, which make them useful for a broad range of applications in various fields. These use-oriented properties are in the first instance determined by the physicochemical properties of nanomaterials, e.g., their composition, size, shape, crystallinity, and surface. In addition, use-oriented properties are also influenced by, or even resulting from, further incorporation of nanomaterials into specific matrices (e.g., composites, food, and cement) or other interaction forms. The use-oriented properties are therefore the basis for applications of MNMs. Functionality has to be optimised specifically during development of nano-enabled products. This aspect is even more important in chemically reactive or complex MNMs (e.g., polymers, metal-organic frameworks), including more than one use-oriented property. The Multifunctional Efficiency [23] adapted from the atom economy concept in green chemistry is a way to maximize the functionality of a MNM by reducing the presence of inactive components, and at the same time to develop a synthesis process as simple and sustainable as possible.

In contrast to maximising functionality, nanomaterials or nanoproducts are expected to neither affect human health nor impact the environment along their life-cycle. Safety is regarded as the reciprocal of risk, which is a measure of the probability that harm will occur under defined conditions of exposure to a nanomaterial or nanoproduct. Aspects of nanomaterials safety have to consider both hazard, which is the potential of a nanomaterial to cause damage, and exposure, which includes that a certain dose encounters human beings or the environment over time. Therefore, safe applications of MNMs or nanoproducts need considerations of their potential hazard and risk arising during the manufacturing process itself as well during their use or disposal. Overall, nanomaterials with a low hazard potential but to which there is frequent or excessive exposure may pose as much risk as nanomaterials, which have a high degree of hazard but to which only limited exposure occurs [24].

**Figure 1:**
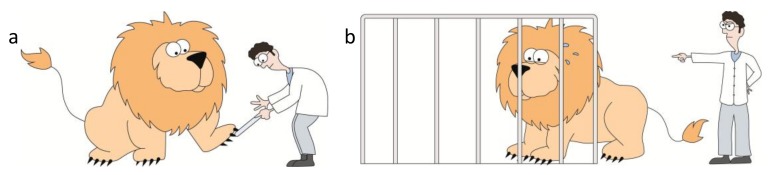
Safe-by-Design strategies: (**a**) Design out hazard and (**b**) Reduce exposure.

This already indicates two main strategies for SbD: the first one is to reduce hazard and the second is to reduce exposure mediated by release of nanomaterials during the life cycle (Figure 1).

The implementation of SbD is based on knowledge of the relationship between designed MNMs properties, their interactions and effects at the cellular and body level, the initiated toxicological response as well as potential release and exposure scenarios. On a comprehensive basis, such information is only available for a small number of MNMs, for example pristine particles from the Organisation for Economic Co-operation and Development (OECD) program and international initiatives. Currently, several EU funded Nanosafety projects have the goal to provide a conceptual foundation to allow drawing relationships between material characteristics, e.g., physicochemical properties and their mode of action regarding toxicity and Adverse Outcome Pathways (AOP) following potential uptake dose. These frameworks and related databases can be used to design tailored MNMs with an optimal balance between functionality and hazard or risk, derived from relevant physicochemical parameters (e.g., elemental composition, size, shape, and surface modification).

## 3. Bottom-Up Approach to Safe-by-Design

In the “Safe-by-Design” concept as developed in NANoREG and ProSafe, the term “Design” is not limited to the property of MNMs, selected and/or modified to have a safer functional product [21,25]. Instead, the term “Design” is applied to the whole innovation process, including production processes, materials, and products. The implementation of this concept, while based on MNMs properties, needs more than the pure scientific knowledge on MNMs safety and functionality to derive proper SbD strategies [26] and to be implemented at the industry level.

### 3.1. The NANoREG SbD Concept

Both the NANoREG and the ProSafe initiatives worked towards the development of a SbD concept for Manufactured Nano Materials (MNM) which could be implemented by industry and taken into account as a reference tool by regulators. For its implementation, the developed concept integrates currently used innovation management processes, risk assessment practices, Environmental, Health, and Safety (EHS) assessment, regulatory affairs, and data handling. It is important to stress that the approach does not substitute the existing frameworks and methods but complements currently used innovation processes.

**Figure 2:**
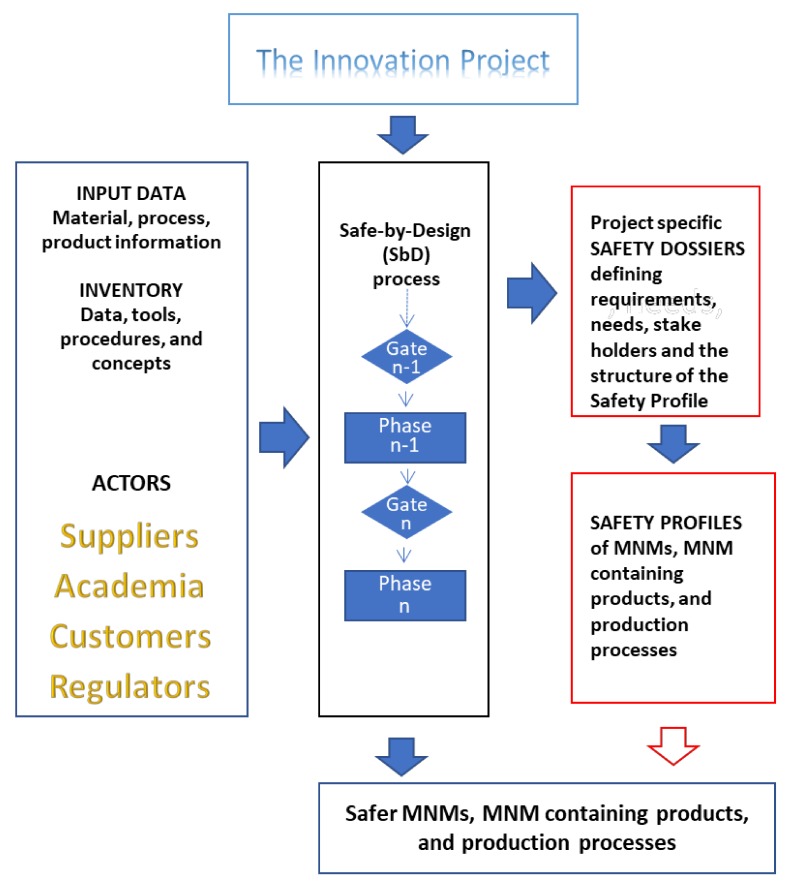
Overview of the SbD implementation process including data inputs, procedures, and the output as Safety Dossier and Safety Profile of the material/ product under development.

The SbD implementation concept, as developed by NANoREG and ProSafe [27], consists of four main elements that compose the overall framework and take into account relevant aspects in data generation, management, and communication (Figure 2).

The four elements are:

(1) the innovation project, including all participating actors;
(2) the safety dossier;
(3) the safety profile; and
(4) the harmonised inventory of the state of the art of SbD protocols and procedures.

The implementation concept clearly differentiates between the SbD process on one hand and the manufactured nanomaterial related data handling on the other. Whereas, the SbD can be applied to different processes, products, companies, and industries, the data is project specific and will be defined in the project specific Safety Dossier and collected under the Safety Profile of the material/product under development.

The objective of the SbD implementation is to transfer the precautionary principle into practical use. This includes precautionary measures and tools for the timely identification of uncertainties and, if possible, their respective risks, at the earliest possible/feasible time in the innovation under development. Uncertainties and risks are not only identified on the material, but also on the potential product reaching the market at the end of the project. In essence, it is anticipating all available information (including reasonable assumptions) and using it as early as possible. Furthermore, it is possible that the result of the application of the SbD concept should focus on risk management options and help to detect knowledge gaps as well as give support to determine the cost of a risk and its effects.

### 3.2. Elements of the SbD Concept

**Figure 3:**
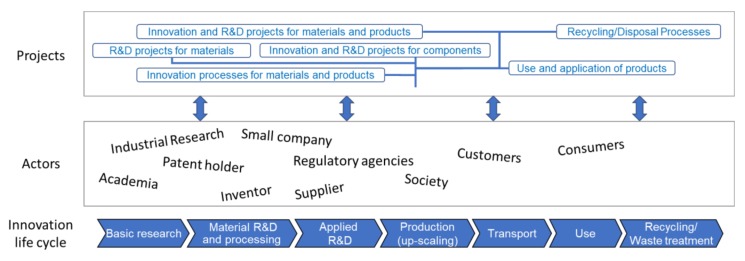
Organization of the innovation project workflow.

The Safe-by-Design implementation concept starts with the definition of a project workflow (Figure 3). This step is very important since here the phases and decision points of a given project will be defined, e.g., at which points within the project one needs to stop and review the data collected to take a decision about the project status. At these points, the innovator will decide if the collected information is promising enough so the project could proceed to the next phase or else, if the collected information indicates reviewing of the product/process before going ahead. In general, a required revision could be suggested by both safety concerns and functionality shortcomings, but also from feedback from the public or regulators, depending on the status of the innovation development.

The structure of phases and gates of a project can be quite complex, with many actors interacting in different phases of the innovation process, especially if we consider models like the Open Innovation. However, also different regulations and standards are applicable to different actors and at different times of the project. Therefore, in our opinion the SbD approach needs to be flexible enough to manage the safety and regulatory data requirements of each small innovation piece and organize the links between actors having different roles.

The functionality and safety assessments are based on data, e.g., on MNMs properties, safety requirements, processes characteristics, products categories, and use profiles. Therefore, the SbD should include a template to identify the right data to collect in a certain phase of the innovation project, in relation to the specific needs. Being the SbD concept developed in a regulatory context, the basis is the regulatory requirements, such as REACH, Biocidal Products regulation, or Occupational Safety information requirements. However, there are other data and information requirements, referring to standards, voluntary certifications, as well as sustainability assessment, etc. These requirements, while not so relevant for regulators, are very important for companies, which are using the “labels” as quality certification toward the customers and consumers.

However, in nanotechnology we always say that the appropriate tools and data templates to assess for example safety or functionality are missing. There is a lot of work going on in different fora (e.g., OECD, International Organization for Standardization, European Commission) to achieve a standardization of methods also for nanomaterials, and to develop better prediction models (see Horizon 2020 project caLIBRAte on model calibration and NanoReg2 on grouping). Therefore, to address this fast growth of tools and methods, we think that a comparable flexible approach must be followed. The SbD data should be collected by using the most advanced tool, that are included in a SbD library to be constantly updated, providing always the best methods to generate and report the information. Such an inventory should include sources and material such as: databases, reference to standard materials, grouping strategies, testing strategies, relevant scientific literature, exposure and toxicity models, Quantitative Structure Activity Relationships (QSARs), etc.

The list of parameters collected (with the appropriate method and tool) to address a specific information need are summarised in the form of a Safety Dossier. The inputs are the selected data requirements (with specification to quantity and quality) at a specific phase of the innovation project. At this stage it is possible to define limit value (regulatory or innovation-related) that could be used to support the decision making at the earliest possible time. For example, if for an application the exposure potential has to be below a certain level, we can set the value into the Safety Dossier and compare it to the measured or simulated exposure data.

The data needs for the Safety Dossier evolve along the innovation project and are specific for the innovation phase under assessment. On one hand, the type of tools and methods that can be used along the innovation chain are selected on the basis of data needs, data quality, and cost. Semi-quantitative approaches like Control Banding are very useful at the beginning of the innovation process, while at more advanced phases (e.g., prototyping) more quantitative experimental data for specific endpoints or exposure scenarios are needed.

On the other hand, there is a multidimensionality of the data needs. As an example, when we are in the idea stage, or when we need to select the MNM of choice, the amount of information is more related to the MNM functionality (e.g., physicochemical properties) and basic toxicological information (e.g., in vitro genotoxicity). At the same time, we think that it would be useful to roughly know in advance what the potential exposure scenarios are for the MNM in the foreseen final product, to identify critical properties that could help us to find the right MNM or the best way to incorporate the MNM in the product. In general, during the innovation process the needs and the accuracy of data will grow, it is in the early phases that in depth knowledge of some aspects is needed. In the final phases, the required data are defined by the applicable regulation.

Once the information is collected and organized, it has to be communicated. The management of the data communication between involved actors is central to have an effective innovation project. There are sensitive data that are restricted to the company (or to a person in the company), but other data needs to be communicated to customers (e.g., labelling information, use instructions, hazard classification) or to suppliers (e.g., exact specifications of the MNMs). Therefore, it has to be possible to “assign” certain information to certain target group/s to make it visible only to them.

The data elaborated in the Safety Dossier are shown in the Safety Profile. The difference with the Safety Dossier is that it includes the evaluation made by the decision makers based on e.g., the regulators feedbacks, the thresholds identified by the innovator, the functionality specifications. There are as many Safety Profiles as phases in the innovation projects and decision points, so the detail (qualitative vs. quantitative information) and the focus (a production process vs. a material) may vary. As a conclusion in the Safety Profile, the relevant information for the different stakeholders will be summarized. Any qualitative and quantitative uncertainty has to be listed in the Safety Profile.

As highlighted in the introduction, a sustainable innovation through Safe-by-Design cannot be achieved without a quality dialogue with other stakeholders and primarily regulators. The next chapter will describe the Trusted Environment concept proposed to enable communication and the exchange of sensitive information.

## 4. Trusted Environments

Innovations in nanotechnology take place in a context of rapid technological progress. It is hard, especially for small and medium sized enterprises (SME’s) where most of the innovations in early stages take place, to keep up with the continuous flow of research and data, relevant for their innovative process. Also, it is not easy to control or reduce the regulatory uncertainties in the innovation process. Parker suggests that innovation processes in general are moving from internal focused “research and development” processes towards more external focused and cooperative processes [28]. The speed in which they are executed is continuously increasing. This is certainly the case with nanotech.

Justo-Hanani [29] found that regulatory uncertainties are (becoming more of) a threat to innovation and therefore to competitiveness and the effectiveness of innovations.

Strategic behaviour of innovative companies may also be analysed from the point of view of game theory. When we consider the decision to share innovation in an innovation process as a game, comparable to the “prisoner’s dilemma”, the strategy of “self-interest” will prevail under most conditions, which means that information will not be shared. However, this could change when conditions are changed for example by introducing rewards/incentives, effective threats, or by changing the conditions of the game, for example by playing a series of games and by giving participants access to the outcome of decisions of co-players.

Based on these assumptions, we conclude that information sharing during the innovation process in nanotech could lead to a more effective innovation process, positive effects on the reputation of companies and nanotechnology, and to “strategic spill over”, the possibility that new or better innovations emerge in the process of information sharing. From the point of view of the regulators, the effectiveness of regulation and the awareness of technological developments may be enhanced, leading to a better preparedness for innovations.

Therefore, effective nanotech innovation in the European Union (EU) will be stimulated if we develop an environment in which information sharing is made easier and safer for the participants. An environment in which both innovators and regulators feel “safe” to share information with regard to innovations. Such an environment can be referred as a “trusted environment”.

### 4.1. Definition of a Trusted Environment

Our definition of a trusted environment is as follows: “An information platform, used to exchange information in a safe and trusted way by innovators and regulators, as a part of the Safe-by-Design approach, consisting of relevant knowledge on manufactured nanomaterials and/or nanotechnologies and governed by an independent organisation”. 

The trusted environment is an internet-based information platform, but not exclusively. Behind the information platform may exist a community of actors (nanotech innovators and regulators) who use the platform to share knowledge or to collect it. Information exchange could also take place by face-to-face meetings between members of this community. An essential precondition of the trusted environment is that innovators and regulators should be able to exchange information rapidly and in a safe or secure way, with regard to their interests. They should be able to control with whom they share a particular piece of information. Innovators should be able to exchange information without fear of losing any competitive advantage or intellectual property and regulators should not have to worry if an exchange of information would lead to unwanted precedents or preferential treatment in regulation or the monitoring of compliance.

The platform could be used in relation to (safe or responsible) innovation processes that may lead to the development of manufactured nanomaterial-based products. “Safe-by-Design” is a process or a way to develop manufactured nanomaterials, and the trusted environment represents a source of information for participants in such an innovation process. Thus, both elements are not only connected, but also reinforcing each other.

To illustrate what we mean by this, we would like to present three potential applications of the trusted environment in relation to the SbD concept.

In the first application, innovators would be able to use the *platform as a database* to find out whether the materials they are developing, are subjected to certain hazards. Any queries or results would have to be confidential. Using the platform would also include sharing information with the platform, so that the database will become bigger and more useful over time.

A second use of the trusted environment would be to use the *platform as an* “*internet forum*” in a process to accumulate and share information on certain topics related to the innovation of nanomaterials. Again, the participation in such an environment should be confidential and to some extent even anonymous, for example by only revealing the role or expertise of a participant.

In the third place, participants could use the *platform as a source of information*. Especially small and medium sized companies would benefit from this use of the platform, using it as a one stop shop for information that has been validated in some way. But at the same time regulators could use the platform as a source of information when they reflect on the needs for future regulation.

### 4.2. Governance and Building of Trust

In our view, the information platform should be governed by an independent body, independent from innovators and from regulators. The most logical design would be that it is a foundation or an association. A big advantage of using the legal identity of an association is that it could be funded by its members. An advantage to the foundation identity would be that its independence can be safeguarded in the best possible way. It will be possible though to find a solution in which both funding and independence are safeguarded.

The organisation itself does not produce information, it merely facilitates the information platform in a technical and in an organisational way. The organisation has an important role in the process of upgrading information to knowledge (validation of information). This should be as much as possible a monitoring and governing role.

The organisation should be permitted to work with these essential, trust building, legal principles:

- it has to be possible to share information in confidentiality, this confidentiality should have a stronger basis than just a non-disclosure agreement;
- it has to be possible to share information in anonymity, meaning that the role of a participant may be visible to other participant but not the name or the exact identity; and
- it has to be possible to make “non-binding statements”, meaning that a participant will not be liable for the statements he or she made during the process of information sharing; this will be particularly relevant for regulators.

Apart from these legal aspects, for the trusted environment to have a real impact on the innovation process, it must be valued and used by innovators and regulators. In the process of design of the trusted environment (ongoing work within the NanoReg2 project), we deliberately use methodologies and techniques of collaborative design, because we think it is crucial to avoid any “not invented here” reactions when developing a “trusted” environment for others. But not just the design process, also the design itself has to be trustworthy.

First of all, the government of the platform must be effective and unbiased. Participants must be given a good reason to participate, one of the best reasons is that they consider it to be valuable when they participate. And then there is a psychological process: if many participate, the burden to join them will become lower, many participants cannot be wrong. So: participants should be stimulated to participate.

## 5. Conclusions

**Figure 4:**
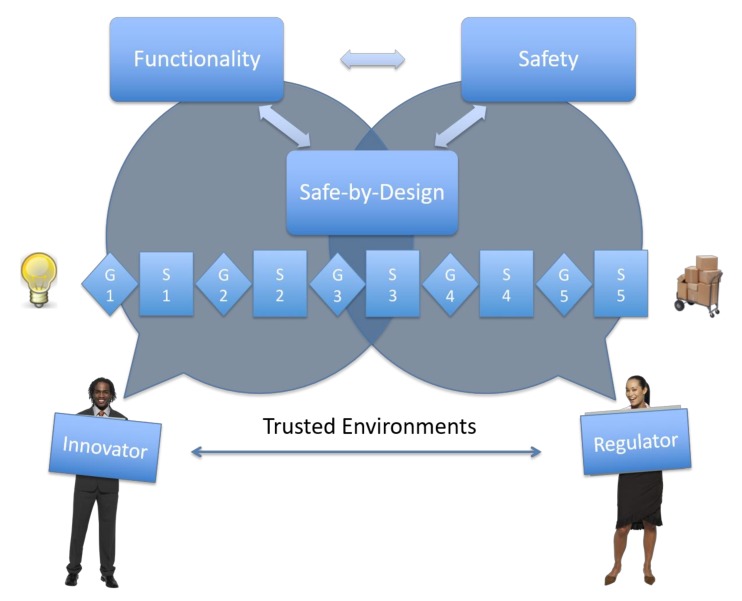
Integrated overview of the relationships between Innovators and Regulators in the Safe-by-Design and Trusted Environment system. Safe-by-Design addresses both Functionality and Safety, while Trusted Environments are the safe lines of communication between the two parts. G: Gates, S: Stage.

The whole SbD concept as proposed in this opinion paper is directly linked to the concept of “responsible (research and) innovation”, and it goes beyond designing less hazardous materials, or a greener product or process. Starting from the (scientific) relationships between the MNMs characteristics and their properties which can be studied at the design stage, the challenge is to implement this knowledge into industrial processes, focusing on applications and uses in a regulatory framework. We think that it is necessary to focus on three aspects of the translation to industry: functionality; safety; and communication. In this opinion paper, we presented two concepts to address these aspects, i.e., Safe-by-Design and Trusted Environments. The integration of functionality, safety, and information exchange, will allow timely and effective high-tech innovation, safer for humans and the environment, that will be accepted and valued by customers and society as a whole (Figure 4). This approach, when implemented, could be one of the main elements of risk governance of nanotechnology. For example, it defines the final goal (functional and safe innovation, economic growth), the information management (safe collection, storage, exchange, and communication), the assessment tools (SbD tools inventory), and a systematic learning process (recursive approach), integrating all stakeholders.

What we see as concrete value for all stakeholders (academia, industry, regulators, and society) is:

- adopting a pragmatic product-oriented Responsible Research & Innovation (RR&I) approach in the whole innovation chain, starting from academia-based start-ups and spin-offs, balancing functionality and safety from the beginning of the innovation project;
- up-to-date centralized inventory of reliable tools and methods for nanomaterial characterization, safety, and sustainability assessment;
- safe and trustworthy information exchange and management system, with a one-stop-shop for pertinent information retrieval;
- reduction of overall costs due to intelligent testing strategy, and faster technological and regulatory screening of promising innovation projects;
- generation of higher quality regulatory safety information;
- structured and trustworthy interaction of innovators with regulators, reducing regulatory uncertainty for companies and allowing faster authorization processes;
- early-on assessment by regulators of the suitability of existing regulations for the innovation;
- timely regulatory development reducing uncertainty for companies and making the development of the innovation sector faster and safer, transferring the benefits to the society; and
- increasing the transparency of the overall process and thus the trust of the public.

However, in our opinion such a complex system cannot be forced through regulation, because it will not make it any more valuable, and it will take years to be implemented. The implementation of Safe-by-Design and Trusted Environments, at least initially, will have to be based on voluntary cooperation of participants. As the saying goes: “You can bring a horse to water but you can’t force it to drink”. It is, in fact, a discussion on the value of participating. Safety by Design may partially be achieved in the context of technology [30], but it will also be a matter of responsible innovative behaviour by both innovators and regulators. However, until there is not the regulatory recognition of the value of Safe-by-Design and Trusted Environments, there is the need to give value to what sums-up essentially as a voluntary approach. What could help is a marketing approach, with a “Safe-by-Design label” or hallmark, to stipulate that participants are innovating in a responsible way. For this to happen, one main issue will be working towards the Standardization of Safe-by-Design, so, all relevant stakeholders will refer to the same issue and follow the same approach.

With the Safe-by-Design processes, combined with the Trusted Environment, Europe may be capable of “steering” innovation processes in the right direction. Going a step further in the implementation of responsible research for consumers and the environment.

## Figures and Tables

**Table 1:** Safe-by-Design (SbD) strategies described in scientific publications (2011–2018).

SbD Strategy	Measure	Source
**Design out hazard (direct and indirect effects of nanomaterials)**	NanoParticle (NP) doping	[1,2]
	Surface passivation	[3,4,5]
	NP coating	
	Reduction of photo-catalytic efficiency	[6]
	Formation of composites	[7,8]
	Surface functionalisation	[9]
**Reduce release**	Adaptation of the processing	[10]
	Selection of nanofiller	[11]
**Reduce bio-persistence**	Carbon NanoTubes (CNT) Doping	[12]
**Testing strategies for safety evaluation**	High throughput screening, alternative testing strategies and biological mechanisms	[13,14,15,16]
**Material characterisation**		[17]
**Identification of risk hotspots for potential SbD approaches**	End of life cycle: thermal decomposition Life cycle assessment	[5,18,19]
**Pilot plant development**	Risk mitigation	[20]
